# Characterization of Dissolved Organic Matter Released from Aged Biochar: A Comparative Study of Two Feedstocks and Multiple Aging Approaches

**DOI:** 10.3390/molecules28114558

**Published:** 2023-06-05

**Authors:** Yan Yue, Leqi Xu, Guitong Li, Xiang Gao, Hongfang Ma

**Affiliations:** 1 Engineering & Technology Center of Electrochemistry, School of Chemistry and Chemical Engineering, Qilu University of Technology (Shandong Academy of Sciences), Jinan 250353, China; yueyan@qlu.edu.cn (Y.Y.);; 2 Yantai Research Institute, China Agricultural University, Yantai 264670, China; 3 College of Land Science and Technology, China Agricultural University, Beijing 100193, China; 4 School of Environmental Science and Engineering, Qilu University of Technology (Shandong Academy of Sciences), Jinan 250353, China

**Keywords:** aged biochar, dissolved organic matter, excitation–emission matrix, fluorescence regional integration, parallel factor analysis

## Abstract

Dissolved organic matter (DOM) plays important roles in environmental ecosystems. While many studies have explored the characteristics of aged biochar, limited information is available about the properties of DOM derived from aged biochar. In this study, biochar obtained from maize stalk and soybean straw were aged using farmland or vegetable-soil solution, as well as soil solution containing hydrogen peroxide (H_2_O_2_). Chemical composition of the extracted DOM from the aged biochar was analyzed via excitation–emission matrix coupled with fluorescence regional integration (FRI) and parallel factor analysis (PARAFAC). Obtained results showed that biochar aged with H_2_O_2_-enriched soil solution had higher water-soluble organic carbon, ranging from 147.26–734.13% higher than the controls. FRI analysis revealed fulvic and humic-like organics as the key components, with a considerable increase of 57.48–235.96% in the humic-like component, especially in soybean-straw-aged biochar. PARAFAC identified four humic-like substance components. Concurrently, the aromaticity and humification of the aged-biochar-derived DOM increased, while the molecular weight decreased. These findings suggest that DOM derived from aged biochar, with a high content of humic-like organics, might impact the mobility and toxicity of pollutants in soil.

## 1. Introduction

Biochar, with large specific surface area (SSA), a well-developed pore structure, and diverse oxygen-containing functional groups, is often used to remediate soils contaminated with heavy metals and organic pollutants [1,2]. Notably, physicochemical properties of biochar, such as surface characteristics, functional groups, and hydrophilicity, can change when incorporated into soil, due to exposure to biotic and abiotic stresses that contribute to biochar aging [3,4]. Research has shown that aged biochar typically exhibits greater SSA, rougher surface morphology, lower acidity, higher ion exchange capacity, and increased aromaticity [4,5]. However, the properties of dissolved organic matter (DOM) released from aged biochar are still insufficiently understood.

DOM, as determined by the measurement of water-soluble organic carbon, is a crucial component of biochar [6,7]. It not only contributes to biochar stability but also supplies nutrients to plants and microorganisms in agroecosystems [8,9]. Furthermore, it plays a critical role in shaping the fate of heavy metals and organic contaminants in the environment [9,10]. The characteristics of DOM released from biochar are greatly affected by pyrolysis temperature [11,12], feedstock [13,14,15], the carbonation process [16], and environmental conditions [17]. However, there are limited studies examining how the aging process influences the properties of DOM derived from biochar, particularly from maize stalk and soybean straw, which are produced in large quantities in China [18]. Therefore, understanding the concentration and chemical composition of DOM derived from aged biochar is essential for accurately assessing its environmental behavior in the soil ecosystem.

Several analytical techniques, including solid-state carbon nuclear magnetic resonance, Fourier transform infrared analysis, ultraviolet–visible absorption spectra, fluorescence regional integration (FRI), and parallel factor analysis (PARAFAC), have been employed to study the properties of DOM [19,20]. Among these, FRI and PARAFAC, which are quantitative methods, are most commonly used for interpreting the fluorescence excitation–emission matrices (EEMs) of DOM in samples from marine, aquatic, and soil environments [21,22,23,24]. FRI analyzes the fractions of DOM by dividing EEMs into five regions: tyrosine and tyrosine-like, tryptophan and tryptophan-like, soluble-microbial-by-product-like, humic-acid-like, and fulvic-acid-like substances, based on the integration of the volume beneath each EEM region [19]. However, FRI is unable to identify some overlapping fluorescence peaks [25]. In contrast, PARAFAC modelling can differentiate specific fluorescent components with similar peaks [26]. Still, it is typically best suited for model datasets with 20–100 samples [27]. As such, combining EEMs with both FRI and PARAFAC modelling might offer a more comprehensive interpretation of the properties of DOM derived from aged biochar.

These things considered, we obtained DOM from aged biochar subjected to various aging processes, allowing for the analysis of its concentration and composition. Consequently, the objectives of this study are as follows: (1) quantify the DOM emanating from the aged biochar sourced from maize stalk and soybean straw; (2) examine the alterations in the DOM derived from the aged biochar, identifying the key or indicative components using FRI and PARAFAC; and (3) elucidate the impacts of differing aging approaches on the spectroscopic properties of the DOM derived from aged biochar.

## 2. Results and Discussion

### 2.1. Impact of Feedstock Type and Aging Method on Biochar Aging Duration and WSOC Content

**Figure 1:**
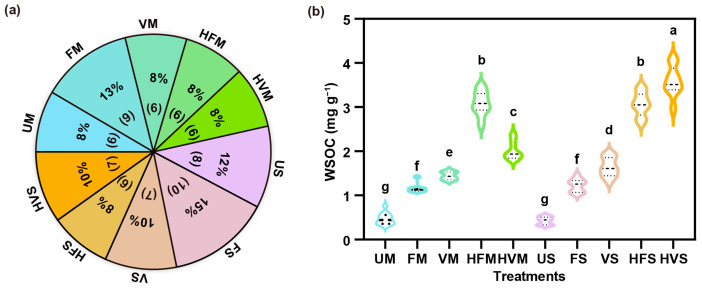
Differences in aging duration (**a**), and water-soluble organic carbon (WSOC) (**b**) in maize stalk and soybean straw biochars subjected to various aging solutions: ultrapure water (UM, US), farmland-soil solution (FM, FS), vegetable-soil solution (VM, VS), and soil solution with hydrogen peroxide (H_2_O_2_) (HFM, HFS, HVM, HVS). Different lowercase letters indicate a significant difference between treatments at the *p* < 0.05 level.

Feedstock type influences the duration of biochar aging when subjected to various aging methods (Figure 1a). For instance, when maize stalk biochar was aged using ultra-water (UM), vegetable-soil solution (VM), farmland-soil solution with H_2_O_2_ (HFM), and vegetable-soil solution with H_2_O_2_ (HVM), each method was applied six times each. In contrast, soybean straw biochar aged with ultra-water (US), farmland-soil solution (FS), and farmland-soil solution with H_2_O_2_ (HFS) underwent 8, 10, and 6 cycles of aging, respectively. Meanwhile, soybean straw biochar aged with both vegetable-soil solution (VS) and vegetable-soil solution with H_2_O_2_ (HVS) underwent seven cycles each. On average, biochar derived from maize stalk and soybean straw experienced 6 and 7.6 aging cycles, respectively. This implies that maize stalk biochar may be more susceptible to aging by soil solution. SEM images supported this conclusion, showing more pronounced damage to maize stalk biochar caused by soil solution (Figure S1a–n). Furthermore, biochar aged using vegetable-soil solution exhibited shorter aging durations than that aged with farmland-soil solution. This could because the higher salt concentration in vegetable-soil solution caused more extensive biochar degradation [28]. SEM results also revealed a greater number of debris particles in biochar aged by vegetable-soil solution (Figure S1a–n).

The aging approach significantly impacted the water-soluble organic carbon (WSOC) content of biochar derived from maize stalk and soybean straw (Figure 1b). For UM, the WSOC content was 0.47 mg g^−1^, which increased by 1.47 and 2.07 times in FM and VM, respectively. This increase suggests that biochar aged by soil solution exhibited enhanced WSOC content, possibly due to the release of soil organic matter adsorbed onto the biochar surface from soil solution [29]. Notably, WSOC content in maize stalk biochar aged by soil solution with H_2_O_2_ further increased by 0.40–1.66 times. This is because chemical oxidation by H_2_O_2_ facilitated the decomposition of inert organic carbon in biochar [30,31]. Comparable trends were observed for WSOC content in soybean-straw-derived biochar. High-resolution SEM images revealed the development of small pores on the surface of biochar aged by soil solution with H_2_O_2_ (Figure S1k–n). This observed phenomenon can be attributed to H_2_O_2_’s potent oxidative properties, which react with organic functional groups on the biochar surface to initiate an ‘etching’ process that creates pores, and to the generation of hydroxyl radicals from H_2_O_2_ decomposition that further interact with and oxidize the biochar surface, promoting the formation of additional pores [32].

### 2.2. Fluorescent Components of Aged-Biochar-Derived DOM Identified through FRI Analysis

**Figure 2:**
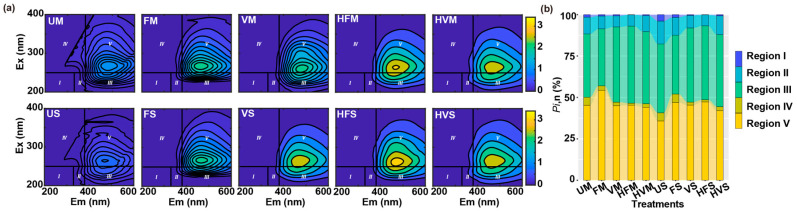
EEM spectral characters (horizontal and vertical lines divide EEMs into five regions (I–V)) (**a**), and distribution of FRI in dissolved organic matter (**b**) for the aged biochar derived from maize stalk and soybean straw (Unit: R.U.).

Fluorescence regional integration (FRI) analysis of dissolved organic matter (DOM) extracted from aged biochar delineates five distinct regions [19]: Regions I and II (excitation wavelengths (Ex) < 250 nm, emission wavelengths (Em) < 350 nm) correspond to simple aromatic proteins, Region III (Ex < 250 nm, Em > 350 nm) represents fulvic-acid-like materials, while Region IV (Ex 250–280 nm, Em < 380 nm) is associated with soluble-microbial-byproduct-like material. Finally, Region V (Ex > 280 nm, Em > 380 nm) is linked to humic-acid-like organics (Figure 2a).

The distribution of volumetric fluorescence among these five regions (i.e., *P*_i,n_) is presented in Figure 2b. For aged biochar, the highest percentages (34% < *P*_III,n_ < 47% and 35% < *P*_V,n_ < 55%) are observed in Regions III and V, respectively. This finding is consistent with the visual analysis of the EEM peak locations within Regions III and V of the delineated regions (Figure 2a). Cao et al. [12] also reported that fulvic-acid- and humic-acid-like fractions predominantly constituted the DOM derived from wood biochar. The aging process resulted in reduced volumetric fluorescence of Region III but increased that of Region V, suggesting an increase in humic-like organics following the aging process (Figure 2b). Xing et al. [33] found that the complexation affinity between Cu(II) and humic-like substances in sludge-based biochar-derived DOM was the highest. This implies that the elevated humic-like organics may contribute to the reduction of mobility and toxicity of heavy metals [9].

**Figure 3:**
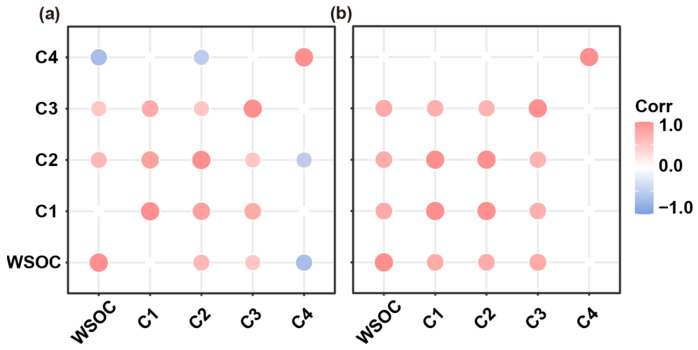
Pearson’s rank correlation analysis of WSOC and normalized EEM volume beneath region “i” for aged biochar derived from maize stalk (**a**), and soybean straw (**b**). Circles represent *p* < 0.05.

Pearson’s correlation analysis was conducted to investigate the relationship between WSOC and the calculated volumetric values in the five defined regions for the DOM (Figure 3). In aged biochar derived from maize stalk, a negative correlation was observed between WSOC and the fluorescence intensity at the excitation–emission wavelengths that fall within Regions I and IV (Figure 3a). A similar trend was observed for DOM extracted from aged biochar derived from soybean straw (Figure 3b). Furthermore, WSOC from soybean-straw-derived aged biochar exhibited a positive correlation with the content of aromatic protein II (Region II), fulvic-like (Region III), and humic-like organics (Region V) (Figure 3b). Distinct differences in functional groups may influence the observed linear correlation between aromatic carbon and fluorescence [19]. This observation was corroborated by FTIR of aged biochar derived from both maize stalk and soybean straw (Figure S2).

### 2.3. Fluorescent Components of Aged-Biochar-Derived DOM Identified by EEM-PARAFAC Analysis

**Figure 4:**
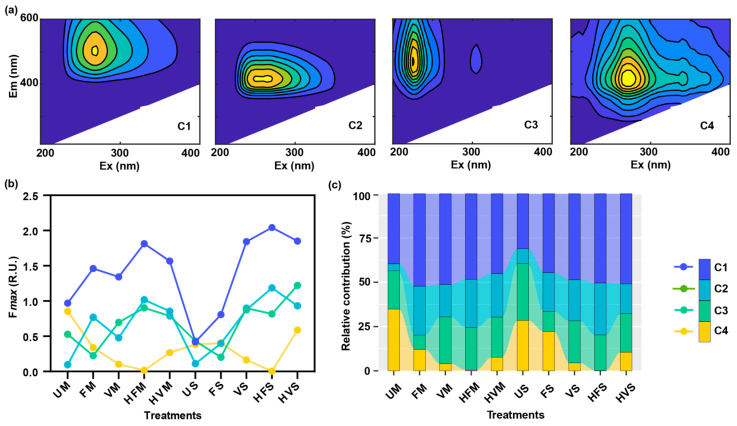
Four fluorescent components identified using PARAFAC analysis (**a**), changes in the *Fmax* values (**b**), and relative contribution (**c**) of four components in dissolved organic matter for aged biochar derived from maize stalk and soybean straw (Unit: R.U.). C1, C2, C3, and C4, humic-acid-like components; R.U., Raman units. Excitation and emission peak positions of three-dimensional excitation–emission matrices for independent components detailed in Table 1.

Four fluorescent components were discerned in the aged biochar derived from maize stalk and soybean straw (Figure 4a, Table 1). A quantitative comparison with the existing components listed in the OpenFluor database [34] indicated that components C1, C2, C3, and C4 are humic-like substances with respective excitation/emission (Ex/Em) maxima of 270/500, 265/415, 225/470, and 270/415 nm [35,36,37,38]. These four components, typically found in terrestrially derived DOM, have been previously reported in biochar samples [39,40].

The aging process influences the fluorescence intensity and composition of DOM in aged biochar obtained from maize stalk and soybean straw (Figure 4b). We observed increases in both C1 and C2, along with a decrease in C4, in biochar aged by soil solution as compared to biochar aged by ultrapure water (Figure 4c). Specifically, C1, which comprised 39.57% of UM, increased by 5.47–12.69% in maize stalk biochar aged with soil solution, while C4, which constituted 34.91% of UM, decreased by 22.75–34.45% in the same aged biochar. Comparable trends were observed in aged biochar derived from soybean straw. Notably, humic-like substances exhibit distinct responses to ultraviolet (UV) light: C1 absorbs light in the UVC and UVA regions, C2 and C4 in the UVC, UVB, and UVA regions, and C3 mainly in the UVC region [26]. These components have previously been reported to facilitate the degradation of organic pollutants under solar or UV light exposure via the generation of reactive oxygen species [41].

**Figure 5:**
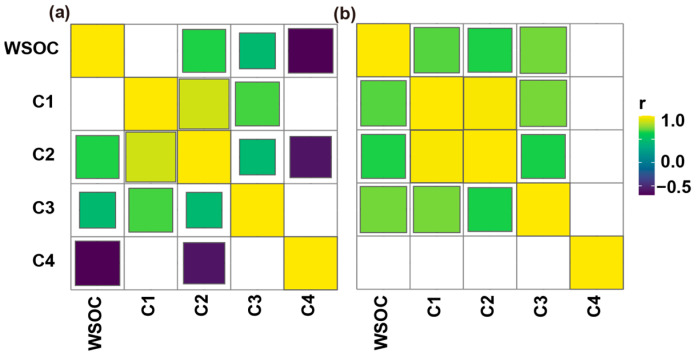
Pearson’s rank correlation analysis of WSOC and *Fmax* values for four identified fluorescent components in aged biochar derived from maize stalk (**a**), and soybean straw (**b**). Squares represent *p* < 0.05.

The correlations between the maximum fluorescence (*Fmax*) values of individual component and WSOC for the aged biochar were evaluated to discern the fluorescent signal component of the aged biochar, based on *p*-values (*n* = 38; Figure 5). For aged biochar derived from maize stalk, C2 and C3 exhibited significant positive correlations with WSOC, while a negative correlation was observed for C4. Similarly, significant positive correlations were observed between WSOC for aged biochar derived from soybean straw and C1, C2, and C3. Evidently, the fluorescent signal components of the aged biochar were primarily impacted by the feedstock type. Notably, C2 and C3 displayed similar changes for aged biochar regardless of feedstock type, suggesting that both C2 and C3 could serve as the signal components for aged biochar.

### 2.4. Spectroscopic Characteristics of DOM Extracted from Aged Biochar

**Figure 6:**
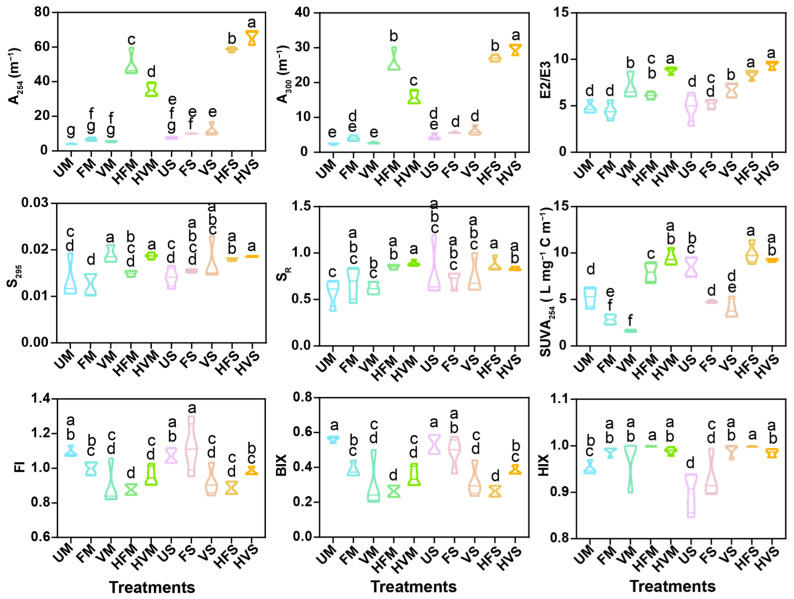
Absorption coefficients at 254 nm (A_254_) and 300 nm (A_300_), absorption coefficient ratio between A_250_ and A_365_ (E2/E3), spectral slope over the 275–295 nm band (S_295_), spectral slope ratio between S_295_ and the slope over 350–400 nm (S_R_), specific ultraviolet absorbance at 254 nm (SUVA_254_), fluorescence index (FI), biological index (BIX), and humification index (HIX) for aged biochar derived from maize stalk and soybean straw. Different letters represent significant difference *p* < 0.05.

Spectroscopic parameters including absorbance coefficients at 254 nm (A_254_) and 300 nm (A_300_), molecular weight (E2/E3), spectral slope (S_295_), spectral slope ratio (S_R_), dissolved organic carbon specific ultraviolet absorbance (SUVA), fluorescence index (FI), biological index (BIX), and humification index (HIX) offer valuable insights into the origins of DOM and their variations due to aging processes and feedstock type (Figure 6). A_254_ and A_300_ exhibited increases by 7.64–11.08 and 5.17–11.62 times, respectively, in biochar aged by soil solution containing H_2_O_2_, hinting at enhanced aromatic fractions and photobleaching of DOM in these samples [22,42]. E2/E3 values for DOM in all aged biochar samples ranged from 4.44 to 9.40, higher values found in biochar aged with soil solutions, suggesting a decrease in DOM molecular weight subsequent to the aging process [14,20]. This observation was consistent with results from S_295_ and S_R_ [11]. SUVA_254_, indicative of DOM aromaticity [14,23,43], showed significant variations. For biochar aged with soil solution from farmland or vegetable land, SUVA_254_ declined by 43.89–68.15%. Conversely, for biochar aged with soil solution containing H_2_O_2_, SUVA_254_ increased to 7.92–9.92 L mg^−1^ C m^−1^, showing a rise by 8.57–80.83% in comparison to UM and US. The marked increase in SUVA_254_ for DOM derived from biochar aged by H_2_O_2_ can be attributed to the polymerization and aromatization of monomers [16]. FI values in the range of 0.87–1.12 designate the origin of the aged-biochar-derived DOM as terrestrial [11]. Low BIX values (below 0.6) in aged biochar allude to the presence of older, decomposed DOM within the biochar [11]. For aged biochar derived from soybean straw, HIX values ascended by 9.43–10.95%, signifying enhanced humification and maturation of DOM [21,44].

### 2.5. PCA Analysis for the Spectroscopic Properties of DOM Extracted from the Aged Biochar

**Figure 7:**
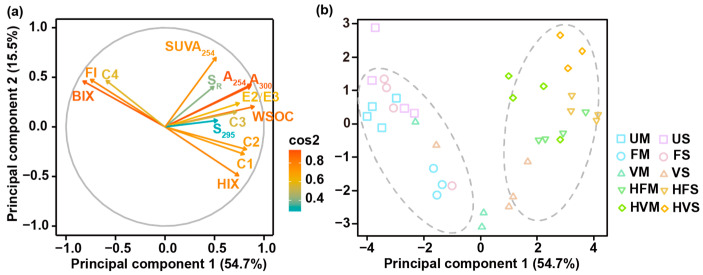
Loading pattern of aged-biochar-derived DOM properties on principal components 1 and 2 (**a**), and score distribution of aged maize-stalk- and soybean-straw-biochar-derived DOM (**b**). Cos2 represents the quality of variations in PCA, with higher values indicating greater contribution of variation to the principal component.

Multivariate statistical analysis, specifically principal component analysis (PCA), was performed to decipher the distinct optical signatures of DOM derived from aged biochar and extract critical information from DOM characteristics (Figure 7). The first two principal components (PC1 and PC2) accounted for 54.7% and 15.5% of the variance, respectively, amounting to a cumulative total of 70.2% of the overall variance (Figure 7a). PC1 is predominantly representative of changes in fluorescence composition, featuring positive loadings associated with A_254_, A_300_, E2/E3, WSOC, C1, C2, C3, and HIX, while negative loadings corresponded to FI, BIX, and C4. In contrast, PC2 had a positive loading for SUVA_254_, implying its representation of the aromaticity of the biochar-derived DOM. The PCA results also suggested intimate relationships between ultraviolet indices and C3, fluorescence indices (FI and BIX) and C4, and HIX with C1 and C2.

The aging processes greatly modified the DOM characteristics of the biochar, resulting in the emergence of two distinct clusters based on these alterations (Figure 7b). The first and second principal components depicted a formation of one cluster consisting of UM, US, FM, and FS, while HFM, HVM, HFS, and HVS comprised the other cluster. Notably, there was a significant shift in PC1 scores from negative to positive for the biochar aged by ultrapure water, soil solution, and soil solution with H_2_O_2_, mirroring the increase in WSOC as indicated in Figure 1b. Further, the samples displayed tighter clustering along the PC2 axis in comparison to the PC1 axis, indicating that the aromaticity of DOM varied greatly with the aging process and feedstock.

## 3. Materials and Methods

### 3.1. Biochar

Maize stalks and soybean straw were washed, air-dried, cut into segments less than 5 cm in length, and then pyrolyzed at 500 °C for 2 h with a heating rate of 10 °C min^−1^ to obtain maize stalk biochar (MB) and soybean straw biochar (SB). This process was based on our earlier research, which indicated that these specific conditions allowed for optimal biochar properties [45,46]. The biochar obtained was sieved using a 2 mm mesh. The corresponding water-soluble organic carbon (WSOC) content for MB and SB was 1.67 and 2.48 mg g^−1^, respectively.

### 3.2. Soil Samples

Topsoil samples, from a depth of 0–20 cm, were collected from farmland soil in Shanyu village (116°45′15″ E, 36°30′33.68″ N), Changqing, Shandong. This farmland follows a traditional winter wheat–summer maize rotation. The soil pH was 6.48, the electrical conductivity (EC) was 115.8 μS cm^−1^, and the water-soluble organic carbon (WSOC) was 264.83 mg kg^−1^. Another soil sample was collected from the topsoil layer (0–20 cm) in a greenhouse of the Vegetable Research and Development Center (118°40′5″ E, 36°57′30″ N), Shouguang, Shandong, where pumpkins were grown. The soil pH and EC values were 6.78 and 314 μS cm^−1^, respectively, and the WSOC was 273.5 mg kg^−1^.

### 3.3. Preparation of Aged Biochar

Soil solution was prepared by mixing soil and water in a 1:2.5 weight-to-volume ratio (*w*/*v*), followed by shaking at 25 °C for 30 min, and then filtered through a 0.45 μm filter. Biochar, either derived from maize stalk or soybean straw, was then added to the obtained soil solutions from both farmland and vegetable soil, at a ratio of 1:10 (*w*/*v*). The mixture was vortexed, shaken at 25 °C for 2 h, and filtered using a 0.45 μm filter, with the filtrate collected for WSOC measurement and the remaining biochar recollected. This entire process was repeated until WSOC levels in the aged biochar remained constant, meaning no significant difference was observed across three consecutive measurements. Biochar derived from maize stalk or soybean straw and aged by farmland-soil solution were denoted as FM and FS, respectively. Similarly, biochar aged by vegetable-soil solution was marked as VM and VS, respectively. In another setup, 30% mass concentration hydrogen peroxide (H_2_O_2_) was diluted with farmland- or vegetable-soil solution to obtain a 3% H_2_O_2_ soil solution. Biochar was then aged using this soil solution, following the previously mentioned steps. Biochar derived from maize stalk and soybean straw and aged by farmland-soil solution with H_2_O_2_ were named as HFM and HFS, respectively, while biochar aged by vegetable-soil solution with H_2_O_2_ were tagged as HVM and HVS, respectively. Fundamentally, biochar subjected to aging via soil solution and soil solution with H_2_O_2_ simulate the normal aging process and the natural oxidation that biochar experiences in environmental conditions [4]. For comparison, biochar, derived from maize stalk or soybean straw, aged by ultrapure water were set as controls and labelled as UM and US, respectively. Each of these treatments was repeated four times.

### 3.4. Analytical Methods

WSOC concentration was measured using a total organic carbon analyzer (TOC-L, CPH, Shimadzu, Kyoto City, Japan). All samples were diluted to less than 10 mg C L^−1^ prior to UV–visible and EEM spectra measurements [47]. UV–visible absorbance spectra were collected between 200 and 800 nm at 1 nm interval using a UV–visible spectrophotometer (TU1901, Persee, Beijing, China). The fluorescence spectra of DOM were obtained using a three-dimensional fluorescence spectrometer (Hitachi F-4600, Hitachi, Tokyo, Japan). This process involved scanning at excitation wavelengths between 200 and 400 nm and emission wavelengths between 200 and 600 nm, with an increment of 5 nm. The speed was 12,000 nm min^−1^ and the peak voltage was 500 V. A scanning spectrum of ultrapure water was used as the control. Surface functional groups of the aged biochar were detected by Fourier transform infrared spectroscopy (FTIR, Thermo Fisher Scientific, Waltham, MA, USA).

### 3.5. Statistical Analysis

Contents of WSOC, the relative fluorescence intensity, as well as *Fmax* value of the individual components identified by PARAFAC modelling for the aged biochar obtained from various aging approaches, were compared using one way analysis of variance via SPSS software (version 26, IBM Corporation, Armonk, NY, USA). The percent fluorescence response in a specific region (*P*_i,n_) was calculated according to the method of Chen et al. [19]. Pearson correlations, corrected using the Benjamini–Hochberg method, between WSOC for the aged biochar and the volume beneath region “i” of the EEM after area normalization were calculated in R software (version 4.2.0) [48].

PARAFAC modelling based on the EEM data was used to analyze the DOM composition. The model was constrained to non-negative values, and the results were validated using split-half analysis and residual analysis [27]. All data and analysis described above were performed in MATLAB 2017a using the DOMFluor toolbox. Detailed comparison and validation of the PARAFAC modelling for the excitation and emission minimum similarity score based on Tucker’s congruence coefficient was, respectively, set 0.94 and 0.95. Pearson correlations, corrected using the Benjamini–Hochberg method, between WSOC for the aged biochar and the identified components from PARAFAC modelling were calculated in R software [48]. DOM spectroscopic parameters including absorbance coefficients at 254 (A_254_) and 300 (A_300_), molecular weight (E2/E3), spectral slope (S_295_), spectral slope ratio (S_R_), water-soluble organic carbon specific ultraviolet absorbance at 254 (SUVA_254_), fluorescence index (FI), biological index (BIX), and humification index (HIX) were calculated, and the above-mentioned parameters are presented in Table S1.

## 4. Conclusions

Feedstock used in biochar production affected the biochar aging process. Specifically, maize stalk biochar was found to be more susceptible to aging by soil solution. DOM released from the biochar significantly increased, particularly when aged by soil solution with H_2_O_2_. Humic-acid-like substances were the main components for the aged-biochar-derived DOM based on FRI and PARAFAC. Additionally, the aromaticity, humification, and maturation of these terrestrially derived DOM in the aged biochar samples increased, while the molecular weight decreased.

However, our study acknowledges certain limitations in the methods employed for aging biochar and the subsequent implications on the findings: (1) environment replication: the aging processes carried out under controlled laboratory conditions may not precisely replicate the wide range of conditions encountered in natural environments; (2) timescale constraints: the accelerated aging processes used in the lab may not accurately reflect the slower, complex processes occurring in nature due to practical time restrictions; (3) biochar type variability: the results derived from maize stalks and soybean straw may not generalize to biochars sourced from other materials; and (4) measurement limitations: utilizing WSOC as an indicator for DOM may not capture all components or nuances of the DOM, nor does it provide insights into the bioavailability or reactivity of the DOM. In light of these findings and limitations, further research should assess potential environmental implications, specifically regarding soil remediation, and weigh the potential benefits and drawbacks associated with DOM release from aged biochar.

## Figures and Tables

**Table 1:** Description of the four components identified by PARAFAC modelling.

Component	Excitation Maxima (nm)	Emission Maxima (nm)	Openfluor Comparison ^1^	Description	References
1	270	500	47	Humic-like	[35]
2	265	415	49	Humic-like	[36]
3	225	470	2	Humic-like	[37]
4	270	415	4	Humic-like	[38]

^1^ Number of studies in the open fluorescence database which previously identified components displaying similar optical properties.

## Data Availability

The data presented in this study are available on request from the corresponding author.

## Supplementary Materials

The following supporting information can be downloaded at: https://www.mdpi.com/article/10.3390/molecules28114558/s1, Figure S1: Scanning electron microscopy (SEM) for maize stalk and soybean straw biochars aged by different solutions: ultrapure water (UM, US), farmland-soil solution (FM, FS), vegetable-soil solution (VM, VS), and soil solution with hydrogen peroxide (H_2_O_2_) (HFM, HFS, HVM, HVS); Figure S2: Fourier transform infrared spectroscopy (FTIR) for maize stalk and soybean straw biochars aged by different solutions: ultrapure water (UM, US), farmland-soil solution (FM, FS), vegetable-soil solution (VM, VS), and soil solution with hydrogen peroxide (H_2_O_2_) (HFM, HFS, HVM, HVS); Table S1: Spectroscopic parameters, indices, and matrices used in this study for DOM characterization.

## Sample Availability

Samples of the compounds are available from the authors.
