# Supplementary material for: Characterization of Dissolved Organic Matter Released from Aged Biochar: A Comparative Study of Two Feedstocks and Multiple Aging Approaches
Source: Molecules. 2023 Jun 5;28(11):4558. doi: 10.3390/molecules28114558 (PMC10254532; doi:10.3390/molecules28114558)
Supplement: Supplementary file 1 [file molecules-28-04558-s001.zip › molecules-2385366-supplementary.pdf]

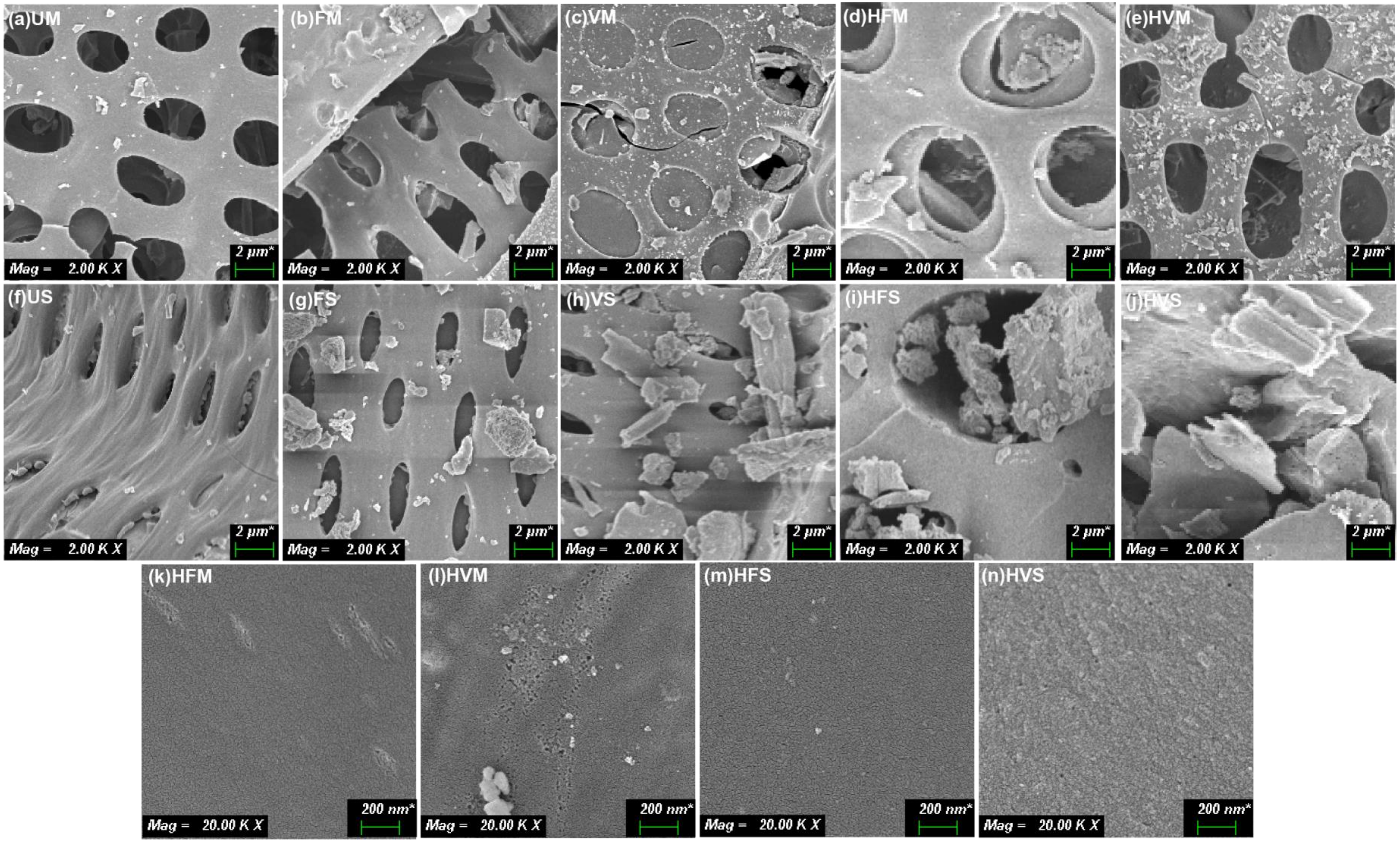
(a)UM
(b)FM
(c)VM
(d)HFM
(e)HVM
(f)US
(g)FS
(h)VS
(i)HFS
(j)HVS
(k)HFM
(l)HVM
(m)HFS
(n)HVS
2 µm*
Mag = 2.00 K X
200 nm*
Mag = 20.00 K X

Figure S1. Scanning electron microscopy (SEM) for maize stalk and soybean straw biochars aged by different solutions: ultrapure water (UM, US), farmland soil solution (FM, FS), vegetable soil solution (VM, VS), and soil solution with hydrogen peroxide (H_2O_2) (HFM, HFS, HVM, HVS).

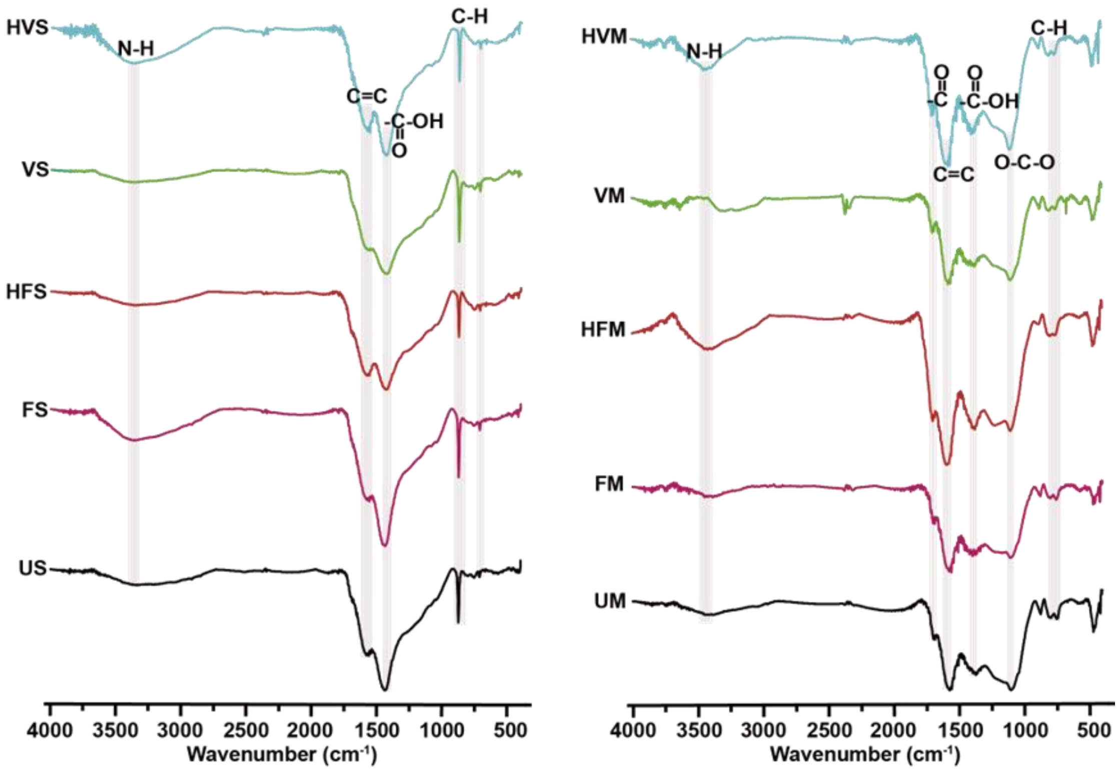

Figure S2. Fourier transform infrared spectroscopy (FTIR) for maize stalk and soybean straw biochars aged by different solutions: ultrapure water (UM, US), farmland soil solution (FM, FS), vegetable soil solution (VM, VS), and soil solution with hydrogen peroxide (H_2O_2) (HFM, HFS, HVM, HVS).

Table S1 Spectroscopic parameters, indices, and matrices used in this study for DOM characterization.

**Spectroscopic parameters**	**Description**
A_{254}	Non normalized absorbance at wavelength of 254 nm [42]
A_{300}	Non normalized absorbance at wavelength of 300 nm [22]
E2/E3	Absorption ratio between the absorbance at 250 and 365 nm [14]
S_{295}	Spectral slope over the spectral band 275-295 nm [24]
S_R	Ratio between S_{295} and the absorption spectral slope over 350-400 nm [24]
$SUVA_{254}$	Specific ultraviolet absorption at 254 nm obtained by dividing A_{254} by water-soluble organic carbon concentration [24]
FI	Ratio of emission intensities at 470 and 520 nm at an excitation wavelength of 370 nm [24]
BIX	Ratio of emission at 380-430 nm at an excitation wavelength of 310 nm [44]
HIX	Area under the emission spectra 435–480 nm divided by the peak area over two spectral bands of 300–345 and 435–480 nm, at an excitation of 254 nm [44]
